# A qualitative study exploring the perceptions of children, parents and school staff towards the development and implementation of school lunch provision within primary schools in the Netherlands

**DOI:** 10.1186/s12889-023-17265-4

**Published:** 2023-11-29

**Authors:** Frédérique C. Rongen, S. Coosje Dijkstra, Tobie H. Hupkens, Monique H. Vingerhoeds, Jacob C. Seidell, Ellen van Kleef

**Affiliations:** 1 Faculty of Science, Vrije Universiteit Amsterdam, Amsterdam Public Health research institute, de Boelelaan 1085, Amsterdam, Netherlands; 2 Food Health & Consumer Research, Wageningen Food & Biobased Research, P.O. Box 17, Wageningen, 6700AA Netherlands; 3 Marketing and Consumer Behaviour Group, Wageningen University, Hollandseweg 1, Wageningen, 6706 KN Netherlands; 4 Amsterdam UMC, location Vrije Universiteit Amsterdam, Public and Occupational Health, De Boelelaan 1117, 1081 HV Amsterdam, Netherlands

**Keywords:** School lunch, School meal, Primary school, School-provided lunch, Organized school lunch, Healthy eating

## Abstract

**Background:**

There is no tradition of serving school lunches in primary schools in the Netherlands. Most children tend to bring their own packed lunch, however these are often nutritionally suboptimal. While school lunch provision can aid healthy eating behavior amongst children, its introduction would constitute a profound change for children, parents and school staff. Therefore, this qualitative study aims to explore children’s, parents and school staffs’ perceptions of both the current lunch situation and the implementation of school lunch provision within primary schools in the Netherlands.

**Methods:**

In this qualitative study we conducted nine interviews with school principals, 98 interviews with children, and held six focus groups with teachers and six with parents at primary schools in two Dutch cities. The data was analysed via iterative coding.

**Results:**

The results showed that most children and parents are satisfied with the current lunch situation, although existing school food policies are not always put in place. Most teachers felt that children had insufficient time to consume their lunch in the current situation. The children were generally positive about the idea of a school lunch, and stressed that it was important to have the ability to choose. While both parents and school staff saw school lunch provision as an opportunity to educate families about healthy food options, they also expressed concern about who would be responsible, as well as the financial and organizational implications of its introduction.

**Conclusions:**

Perceptions of children, parents and school staff about a school provided lunch are mixed. A complex intervention such as a new school lunch program is difficult to envisage for all parties involved and more research is needed regarding the effects, organization, logistics and the costs of school lunch provision in the Netherlands.

**Supplementary Information:**

The online version contains supplementary material available at 10.1186/s12889-023-17265-4.

## Background

In many parts of the world, including in the Netherlands, most children consume too much sugar-sweetened beverages, deep-fried foods, sweet and savory snacks, while, simultaneously, consuming insufficient fruit, vegetables and whole grain products [1, 2]. It is well-established that the eating habits established in childhood are typically carried forward into adulthood [3]. This means that childhood is a critical time period in which children can learn healthy eating behavior. In addition to parenting, school meal programs have also been shown to play an integral role in shaping children’s diets. A review of seven studies that measured the lunchtime nutrient intake among 5–11 year-old English pupils showed that the nutritional quality of homemade packed lunches was inadequate compared to school-provided meals [4]. Similarly, a randomized controlled trial with 8–11 year-old Danish children showed that the overall dietary intake improved when habitual homemade packed lunches were replaced with school-provided meals [5]. Another study demonstrated that serving a healthy school lunch improved the reading performance of 8–11 year-old Danish children; however, there was no enhanced influence upon their concentration compared to the usual homemade packed lunch [6].

Primary schools represent a promising setting for dietary interventions, insofar as children spend more time at school than they do any other environment outside the home [7]. This is reflected in the fact that the eating behaviour of children has been found to be strongly influenced by their school friends and teachers [8]; indeed, seeing their friends try new foods or eating together with their teachers has been shown to stimulate healthy eating routines [9, 10]. Furthermore, national dietary school interventions also have the potential to reach children from all socio-economic backgrounds, thereby potentially reducing the observed socio-economic inequalities in dietary intake [11, 12]. This is important because studies across a wide variety of countries have shown that children of parents with lower socio-economic position (SEP) appear to consume unhealthier diets than children with parents of a higher SEP [13–15].

In contrast to a number of other European countries, such as the UK, Finland and Sweden, there is no tradition of a national school meal program in the Netherlands [16–18]. Generally speaking, children either eat their lunch at home or bring their own packed lunch to school, which commonly consists of sandwiches and a drink. Therefore, children tend to consume very little fruit or vegetables during lunch. Furthermore, children who remain at school during lunch have been found to consume statistically significantly more sugar-sweetened beverages than children who eat their lunch at home [19]. Over the past decade, an increasing number of schools have shifted from giving children the option to eat their lunch at home (traditional timetable) towards introducing a mandatory lunch break at school (continuous timetable) in which children eat their homemade packed lunch. This shift towards a continuous timetable provides an opportunity to introduce a healthy school lunch, and, in turn, could constitute an effective intervention through which to facilitate healthy eating behavior amongst primary schoolchildren from all socio-economic backgrounds.

Undoubtedly, the introduction of a healthy school lunch in the Netherlands would represent a profound change for children, parents and school staff alike, and thus it is essential to take their perspectives into account when examining the development and implementation of a healthy school lunch program. Indeed, several studies have established that the involvement and critical input of stakeholders notably increases the chances of a successful implementation [20]. To garner more support from these various stakeholders, it is of paramount importance that a school lunch is tailored to the specific wishes and needs of children, parents and school staff alike. However, to the best of our knowledge, no studies in the Netherlands have investigated these stakeholders’ perceptions of introducing a national healthy school lunch program. Therefore, this qualitative study aims to explore the perceptions of children, parents and school staff towards both the current school lunch system and the development and implementation of school lunch provision within primary schools in the Netherlands.

## Methods

### Study design

This study is part of the larger Dutch research project “the Healthy School lunch” [21]. This project aims to encourage healthy eating behavior amongst children at primary schools by offering a healthy school lunch, which is based on the Dutch guidelines for a healthy diet. Due to the exploratory nature of the present study, a qualitative research design, namely an inductive approach, was considered the most appropriate research design [22]. We conducted semi-structured interviews with children, school principals, as well as conducting focus group discussions with parents and teachers, in order to gain in-depth insight into the experiences, perspectives, opinions and beliefs of the participants [23]. For the purposes of ensuring consistency across the individual and focus group interviews, both adhered to the same semi-structured interview format and utilized the same interview guide. All interviews and focus group discussions were conducted between March and September 2017, and took place in two Dutch cities: Amsterdam, which is a large city with around 854,000 inhabitants, and Ede, which is a decidedly smaller city with around 115,000 inhabitants. The Social Ethical Committee of Wageningen University (the Netherlands) approved the study protocol.

### Participants and recruitment

A purposive sampling method was used to recruit primary schools to take part in the study [24]. Most of the schools in Amsterdam (206 of 221), along with all of the schools in Ede that were known to have an interest in nutrition (9 of 41), were approached by email to inform them about the study. Follow-up phone calls were then made until we had a sufficiently representative sample of schools from a range of socio-economic backgrounds, neighborhoods and school sizes in both cities. Given that the decision as to whether a school would participate in the study was up to the principals, the first step was to interview the principals. Ultimately, they were free to choose which part(s) of the study they wanted to participate in, that is, the focus groups with the teachers or parents or interviews with the children. The principal then invited teachers to take part in the focus groups through email. Children were recruited through an information letter that they would subsequently pass on to their parents, which both stated the purpose and procedures of the study and passive informed consent (‘opt out’) was obtained. Prior to the interview, children could also indicate whether they wanted to participate. Parents were recruited via the same letter as their child, which asked them to express their interest in participating in a focus group. Parents were also recruited by staff when dropping off or picking up their children at school. Principals, teachers and parents gave their consent orally prior to their participation.

Nine semi-structured interviews with school principals were conducted, of which four decided not to participate in other parts of the study. Two principals gave their approval to conduct interviews with children and focus groups with parents and teachers at their schools, one principal allowed us to conduct focus groups with teachers and interviews with children, one principal gave permission to hold focus groups with teachers and parents, while one principal agreed that we could conduct focus groups with parents and interviews with children. Overall, we conducted six focus groups with teachers (n = 15 teachers; Table 1), six focus groups with parents (one father and 32 mothers), and 98 semi-structured duo or trio interviews, which included 197 children aged between 5 and 12 years-old. The children comprised a range of different educational groups: 10 children were from educational group 3 (6–7 years-old), 36 children were from group 4 (7–8 years-old), 34 children were from group 5 (8–9 years-old), 36 children were from group 6 (9–10 years-old), 42 children were from group 7 (10–11 years-old), while 38 children were from group 8 (11–12 years-old). The schools were located across five different neighborhoods in Amsterdam and two different areas in Ede. Five schools adopted a continuous timetable and four schools adopted a traditional timetable.

**Table 1:** Overview of participants

School number	city	Timetable^1^	SES of the neighborhood	principals	teachers	parents	children
1	Amsterdam, South-east	Continuous	Low	1	3	7	59
2	Ede, North	Continuous	High	1	4	16	52
3	Ede, South	Continuous	Low	1	-	-	-
4	Ede, South	Traditional	Low	1	2	-	48
5	Ede, North	Continuous	Low	1	-	-	-
6	Amsterdam, West	Continuous	Low	1	6	9	-
7	Amsterdam, West	Traditional	Low	1	-	-	-
8	Amsterdam, Central	Traditional	High	1	-	-	-
9	Amsterdam, East	Traditional	High	1	-	1	38

^1^ continuous timetable with a mandatory lunchbreak at school. Traditional timetable children have lunch at home or bring a packed lunch from home to school. SES: socioeconomic status

### Data collection

We developed two interview guides (for children and school staff) and one focus group guide (for parents), which is in accordance with Creswell’s recommendations [24]. These guides began with several short questions about the socio-economic background of the participants (e.g. age, number of children), followed by open-ended questions about both the current lunch situation (e.g. content, preparation, food policy at school) and the introduction of school lunch provision (e.g. organization, challenges, benefits) (Table 2). Probes and follow-up questions were used to encourage participants to talk, provide concrete examples and elaborate on their ideas and opinions. Upon the conclusion of the focus groups and interviews, participants were asked about any topics or issues that had not been raised, but which they felt were important to include. The interview guides were pilot tested and evaluated by the seven interviewers. Based on the pilot test, the questions were slightly adjusted. An interview protocol was developed to ensure that our seven (trained) interviewers applied the interview guides equally.

**Table 2:** Interview – and focus groups guide for children, parents and school principals

Topic	Sample questions
***Children***	
*Part 1: Introduction and welcome*	
- Explanation of discussion topic and rules - Talking to make them familiar	Could you tell me how old you are and what you like to do when you get home?
*Part 2: The current situation of lunch during school days*	
- Questions about where and what they eat during and after school	What are you typically eating during lunch? Are there rules at school about lunch?
*Part 3: Preferences regarding current lunch*	
- Questions about preferences of current lunch - Questions about what they think is healthy of current lunch	Do you like what you eat during lunch? What do you think is healthy to eat and drink for lunch?
*Part 4: Perceptions about lunch at school*	
- Questions regarding different aspects of a school lunch	What do you think of a school lunch? Would you like to help preparing a school lunch? How much time do you need to eat and how much time do you want to play outside?
***Parents***	
*Part 1: Introduction and welcome*	
- Explanation of discussion topic and rules - Introduction and ice-breaking questions	Could you tell me something about your children, how old are they and in which grade?
*Part 2: Current lunch situation*	
- Questions about what their children eat during and after school	Do your children eat their lunch at school or at home? What does your child eat for lunch? Are there rules at school and/or at home for lunch?
*Part 3: Perceptions about a healthy school lunch*	
- Questions regarding different aspects of a school lunch	What do you think of a school lunch? How does the organization of a school lunch look like? What will be the biggest challenge for implementing a school lunch?
***School principals and teachers***	
*Part 1: Introduction and welcome*	
- Explanation of the study	Could you tell me something about your role within the school?
*Part 2: Current lunch situation*	
- Questions about what children eat - Questions about current lunch situation	Do you know what children usually eat for lunch? Are there any rules for lunch at school?
*Part 3: Perceptions about a healthy school lunch*	
- Questions regarding different aspects of a school lunch	What do you think of a school lunch? How does the organization of a school lunch look like? What will be the biggest challenge for implementing a school lunch? What do you need as a school to provide a healthy school lunch? What do you think of using a hostess during lunch?

Prior to the start of the interviews or focus groups, information about the aims of the study, duration of the interview, anonymity and confidentiality were provided. The importance of gaining insight into their opinions, experiences and ideas was emphasized. The children were interviewed primarily in pairs in a quiet and private setting in their school. The average duration of the interviews with children was 17 min (ranging from 9 to 33 min). Interviews with principals were conducted either face-to-face at school or by telephone, with the average duration being 32 min (ranging from 22 to 66 min). Focus groups with parents and teachers were conducted face-to-face in a quiet and private setting in the school. The average duration of the focus groups with both parents and teachers was 50 min (ranging from 25 to 77 min) and 40 min (ranging from 14 to 52 min), respectively.

### Data analysis

All interviews and focus group discussions were audio recorded and transcribed verbatim. The transcripts were then analyzed through a process of iterative coding. The first transcripts were coded independently by multiple researchers (FR, TH and CD) using qualitative software (ATLAS.ti) and discussed afterwards [25]. The topic list was adjusted after coding the transcripts until no new topics emerged. Overall, 581 codes were created for the children, 109 codes for the parents, and 76 for the teachers and principals. Specific quotations were chosen to represent the emergent themes and categories. All quotations were anonymized using unique identifiers (P1, P2, etc.). The findings were supported by quotations from the interviews, which were translated from Dutch into English.

## Results

The results of the interviews and focus groups were organized into two categories: the current lunch situation and school lunch provision. With respect to the *current lunch situation*, the following main themes emerged: general satisfaction with the status quo concerning school lunch; healthy school food policies are not always in place or implemented; and insufficient time for children to eat their lunch. Regarding *school lunch provision*, the following main themes emerged: who is responsible for healthy eating habits; school lunch provision as an opportunity to provide equality and healthy eating habits; practical concerns and benefits of a school lunch; control over food choices during lunchtime; and financial contributions.

### The current lunch situation

#### General satisfaction with the status quo concerning school lunch

Almost all parents had children that attended schools with a continuous timetable, whereby children eat their packed lunch at school rather than going home, which they were very satisfied with. One reason for this satisfaction is that parents stated they no longer had to rush to pick up their children at school, take them home, eat a sandwich and then rush them back to school.

Children and parents also expressed that they usually brought, or provided in the case of parents, a packed lunch to school, which contained bread with a sweet or savory topping and milk, water or a sugar-sweetened beverage. Almost all the children and parents stated that they were satisfied with the current content of the lunch. Most children gave no further explanation for this—they simply liked it. Other reasons as to why the children were satisfied included knowing what was inside the lunchbox (disliked trying new things), variation in their lunchbox (having something different each day), as well as the opportunity to exchange lunch items with other children.

*Child (8 years-old) school 1: “Well, I always like everything, well actually I’m just happy about it. Well, look, I’m completely used to it. I don’t really like new products.“*

Children who were less satisfied with the current content of their lunch expressed that they would like different products, such as white bread instead of brown or another type of fruit. Several children were jealous of the lunches other children brought to school.

Most of the parents and children were also satisfied with the current organization of lunch. Some children prepared their own lunch, while others could choose what they wanted and have their parents prepare it for them. Most parents listened carefully to their children’s food preferences while preparing their lunchboxes. They mentioned that parents were responsible for taking care of their children. While very few parents perceived preparing their children’s lunches to be either problematic or stressful, some did face difficulties in terms of striking the right balance between selecting a healthy product that their children still considered to be tasty. They frequently perceived that when children did not like a certain food or drink, they would not consume it at all. This led parents to search for alternatives that were liked by their children, while, simultaneously, being ‘not very bad’ as one parent put it.

*Mother school 2: “I gave him milk before, but he doesn’t like it when it’s in his cup, because then it’s not so cold anymore. And if he gets that packaged, long-life milk, then he doesn’t drink anything at all. So, that is quite difficult, to find something that you feel is not very bad for him and that he will drink.”*

Some of the teachers noticed differences in what children brought to school. For example, some children had an unhealthy lunchbox containing white bread, cookies or whatever other snack was on offer at the supermarket. However, some teachers indicated that it was up to the parents to decide what to give their children for lunch. Conversely, school principals indicated that they had no insight into what children consumed for lunch.

#### Healthy school food policies are not always in place or implemented

Half of the principals indicated that their school has a healthy food policy in place, which state, for example, that children can only eat fruit and/or vegetables during the morning break, and can only eat a sandwich and drink water or milk for lunch. The remainder of the principals indicated that although their schools did not have a specific food policy in place, they actively stimulated children and parents to bring healthy products to school for lunch.

With respect to those schools that lacked a food policy, parents indicated that there were no strict rules about what they needed to pack for their children, but that the school did advise them not to pack something sweet. Within those schools with a food policy, most of the parents were satisfied with the food policy, particularly the policy about drinking water instead of sugar-sweetened beverages.

*Mother school 1: “What I hear, healthy food, healthy lunch. The snack at 10 am is fruit and water. I’m also glad that the school has abolished those packed soft drinks. Everyone just brings water, which makes me satisfied. At home my children also drink more water.”*

However, parents with children in schools with food policies cited different experiences concerning the degree to which teachers stuck to the rules. Some parents indicated that some teachers did not enforce the policy and let children consume sweet foods and drinks, while others were stricter and confronted parents about the unhealthy products.

Most of the children were aware of the school food policy and mentioned that there were strict rules for what they could or could not bring to school. However, some children still thought it would not be a problem to bring something less healthy to school even when a school food policy was in place.

#### Insufficient time for children to eat their lunch

Most of the principals and teachers identified that the lack of a proper lunchtime in the current lunch schedule was a problem. Five out of nine schools had a continuous timetable with a lunch break typically ranging from 10 to 15 min; however, this break was also part of the educational time, which meant that children had to read a book or watch an educational television program while eating their lunch. After this lunch break children played outside for 30 min. Teachers acknowledged that most children did not have enough time to consume their lunch during lunchtime. Some children could bring their sandwich outside, but others would put it back in their lunchbox. Indeed, the teachers indicated that they often had to sacrifice their own lunch breaks, which was experienced as negative and as contributing to higher levels of work-related stress.

*Teacher school 2: “Yes, fifteen minutes is on the schedule, fifteen minutes to play outside and fifteen minutes to eat lunch, but we usually run a little late.“*

The other four schools had a lunch break that lasted 60 min, whereby children could go home or stay at school for lunch. If children had their lunch at school, they had 30 min to consume their lunch and 30 min to play outside. All principals and teachers, irrespective of the school schedule, felt that having sufficient time was an essential requirement for children to be able to eat without overly rushing.

The children were divided over how long it took to consume their lunch. Some children said that it typically took five minutes for them to consume their sandwich, while others said it was closer to 15 min and that this was too short.

### School lunch situation

#### Who is responsible for healthy eating habits?

A key theme that emerged when explaining the idea of a school lunch was who is ultimately responsible for healthy eating (habits) amongst children. Divergent opinions around the question of responsibility, in turn, led to different perceptions towards school lunch provision. Most school principals considered that although they could help to encourage children to eat healthily, ultimately it remained parents’ responsibility to provide their children with a healthy lunch. They neither wanted to take this responsibility away from parents nor wanted this responsibility thrust upon them. Some teachers and principals said that they merely wanted to focus on teaching children the obligatory/compulsory subjects, such as maths, history and geography.

*Principal school 3: “I think that parents are ultimately responsible for their children, and I think there should be an awareness among the parents. They should think ‘I need to provide healthy food’. I don’t think we should do that as a third party, because then we would be taking the responsibility away from parents, and that can make them very complacent.”*

#### School lunch provision as an opportunity to provide equality and healthy eating habits

Children, parents and teachers all expressed that a school lunch would provide an opportunity for all children to be offered the same lunch, as well as acknowledging that it would potentially encourage children to try new types of food.

*Mother school 2: “It seems to me to be an advantage if everyone gets the same and that they see that others are eating it as well. Maybe they are going to try other things. At home, they say I do not want this. While when they see other children eating it, they might too.”*

There were children who explicitly mentioned that they would no longer be jealous of other children if school lunches were provided. Moreover, teachers thought that the observed differences in the content of lunchboxes would diminish, while several teachers also emphasized the benefits of everyone eating lunch together.

Most parents believed that school lunch provision also provides an opportunity to teach children about healthy food choices, as well as showing them how to prepare/cook healthy meals. They indicated that when children prepared their own food, they were more willing to eat it, even if it was relatively new for them. Some children thought that a school lunch would help both them and their classmates to eat healthier. The children also indicated that they were incredibly enthusiastic about helping to prepare the school lunch.

#### Practical concerns and benefits of a school lunch

Teachers and principals highlighted various practical concerns related to the implementation of school lunch provision. Above all, serving a healthy school lunch, in conjunction with the daily logistics involved, was considered to be an unacceptable burden to place on school staff and teachers. One of the main concerns expressed by principals and teachers was that it would increase teachers’ workload. Other practical concerns pertained to the fact that schools were not suitable environments for preparing lunches (e.g. missing kitchen facilities).

To minimize the burden on teachers, the interviewers proposed using either additional support staff or volunteers. All teachers responded positively towards the idea of support staff helping during lunchtime, in order to minimalize the burden on them. These support staff could be trained employees or parents who volunteer to help during the lunchbreak. However, most of the principals expressed reluctance towards the idea of support staff, on the grounds of finances, organization and competence. Moreover, they indicated that recruiting enough parents to assist with school lunches would also be time-consuming, while hiring a trained employee would entail costs the school could not afford.

*Teacher school 6: “People really need to be hired for that and come to take care of it at school, because to leave that to teachers … I think that will cause too much work pressure.”*

Children were divided in their opinions about a healthy school lunch. Most of the points raised were positive and of a practical nature. Some children believed that an organized healthy school lunch would save both them and their parents time in the morning, not to mention saving money.

*Child (10 years-old) school 2: “I think it’s a good plan. The good thing about it is that you don’t have to prepare lunch before you go to school, so you can sleep a little longer or watch television. Then my mother does not have to do so much work and we would be able to go to school sooner.”*

Parents expressed both positive and negative opinions towards a healthy school lunch. The main positive was that it would provide additional time in the morning and reduce stress. However, some parents expressed concerns over whether children would eat enough if there was a hot school-provided lunch.

#### Control over food choices during lunchtime

Almost all children stressed the importance of being able to choose the content of their lunch. Children who held more negative views towards school lunch provision were worried about their lack of control over their own food choices, and the fact that they may not like the different food and drink options available. Parents expressed similar concerns about the range of products that would be provided during school lunches. Several parents noted that their child was a picky eater and that they were worried they would not eat anything from a healthy school lunch. Other children reported being overly conscious of whether people washed their hands and adhered to food safety guidelines, which is why they preferred to eat their own lunch prepared by their parents. Other concerns from parents pertained to how the school would manage allergies and religious dietary restrictions.

#### Financial contribution

Most of the principals indicated that they lacked the budget for school lunch provision, while asking parents to make a financial contribution was not a viable option. They noted that various parents at their schools were facing financial difficulties and could thus not financially contribute. Other principals mentioned that parents probably would contribute to school lunch provision, but that this was dependent on the total cost.

Almost all parents stated that they were willing to contribute financially to school lunch provision, arguing that there were also expenses related to packed lunches. Parents perceived that if school lunches were to be implemented, then naturally they would contribute financially. However, parents indicated that the contribution towards school lunches should not exceed their current packed lunch expenses.

*Mother school 2: “Yes, but how much? I wouldn’t mind paying something, but if it exceeds a certain limit, then we’re better off doing it ourselves. A few euros a day is fine, but five euros is too much for me.”*

Furthermore, parents stressed that it was important that school lunches offer something more (such as, for example, a bowl of soup) than homemade packed lunches, otherwise they saw little benefit of school lunch provision and would rather prepare lunch for their child.

## Discussion

This study has explored children’s, parents and school staffs’ perceptions of both the current lunch situation and the development and implementation of school lunch provision within Dutch primary schools. Overall, almost all of the children and parents were satisfied with the current organization and content of the lunch, despite the fact that the time allocated for the lunch was deemed to be too short. Consequently, they felt no strong need for school lunch provision. There were divergent opinions regarding the development and implementation of school lunch provision. Most children were positive about the prospect of school lunch provision. However, not all parents and school staff believed that schools should be responsible for children’s healthy lunch habits. Specifically, school staff were worried that school lunches would increase their workload, alongside being financially unfeasible. However, all of the stakeholders recognized the positive opportunities offered by school lunch provision, namely the fact it would give every child access to the same healthy food options, as well as teaching them about healthy food, and making healthy eating a habitual and shared experience.

The parents in this study indicated that they all encountered dilemmas when preparing their children’s lunchboxes: on the one hand, they were deliberating about what was the most healthy food choice, while, on the other hand, they worried this would conflict with their children’s ideas of a tasty lunch. Previous research has shown that parents feel that schools can play a role in fostering healthy eating habits [26]. Indeed, some schools already contribute to healthy eating habits by implementing healthy school food policies and regulations regarding what foods and drinks can be consumed in school. However, this study showed that parents experienced differences in terms of how teachers enforced these food policies, with some being stricter than others. This is in line with other research, which showed that teachers faced difficulties enforcing food policies due to unclear definitions of what constitutes healthy and unhealthy foods [26]. In the present study, the introduction of a healthy school lunch was discussed from the perspective of whether it is the school’s or individual families’ responsibility to foster healthy eating habits. School staff were hesitant to take responsibility for feeding children healthy food, on the grounds that this potentially conflicted with their responsibility to provide good education to their students and ensure a positive working environment for their staff. Teachers and principals mostly felt that parents were primarily responsible for providing a healthy lunch and that they had no wish to take over this responsibility. This position about whose responsibility it is ultimately to ensure healthy eating at school constitutes a key barrier that must be considered when introducing a healthy school lunch program. Above all, this requires working towards an alignment of interests and establishing a shared sense of responsibility between schools and parents [20].

Our study has shown that children, parents and school staff have both varying opinions but also shared ideas regarding the development and implementation of school lunch provision. Essentially, they all see the benefits of school lunch provision. Children were generally positive about its implementation. A recurring theme in both parents and school staffs’ accounts was that a healthy school lunch represented an opportunity to teach children about healthy food choices; however, they also shared concerns about the financial and organizational implications of school lunch provision. Other countries around the world have already shown how to effectively organize and implement school lunch provision [27]. Moreover, a study in the Netherlands investigating the implementation of a ‘’healthy primary school of the future’’ also showed that a school lunch can be successfully implemented [28]. A chief concern from teachers and school principals pertained the consequences of school lunch provision for their workload. This is important because the issue of high workloads among Dutch teachers is already the subject of considerable discussion [29]. Undoubtedly, the implementation of school lunch provision does involve multiple logistical components, such as employee support, finances and provision of suitable kitchen facilities within schools. Therefore, it is of paramount importance to consider these organizational and logistical variables when developing and implementing school lunch provision.

This study raises some important implications for the implementation of school lunch provision in the Netherlands. First, it is important to build an alignment between who is ultimately responsible for providing school lunches and fostering healthy eating habits amongst children. Currently, more research is needed into how best to develop support for the implementation of a healthy school lunch program amongst a broader group of parents. Secondly, it proved to be difficult for the participants to form an opinion about the concept of school lunch provision, insofar as there is no tradition of this in the Netherlands, and, as such, they have no relevant experience of what this would look like. Product development literature informs us that more reliable opinions can be obtained when people are able to provide input into more concrete and visualized ideas [30]. In order to overcome this problem, future research should provide parents, school staff and children with different detailed conceptualizations of school lunch provision, and ask them which concept they prefer. With respect to these concepts, it is instructive to keep in mind that the children in this study stressed that it was important that they had a choice. This is in accordance with other research which stated that children want the opportunity to choose, and that, in this respect, children are the key informants about precisely what motivates their eating habits [31]. Thirdly, school lunch provision is incompatible with the current school timetable. The teachers in this study stressed that children have insufficient time to eat their packed lunch, let alone to be able to sit down for an extensive school lunch. Changing the school timetable would be a lengthy process involving various parties, and, hence, will take time. However, the Dutch “healthy primary school of the future” project showed that it is possible to make changes in the timetable of primary schools. Specifically, they extended the school day to make more time for eating lunch and engaging in other health-related discussions [32]. Fourthly, the current facilities within most primary schools in the Netherlands are not suitable for preparing and serving a proper lunch. Hence, before implementation can be considered, it is first necessary to explore different possibilities, including catering services and kitchen facilities. Alongside this, the costs of these different possibilities must be explored, due to the fact that a lack of financial resources was cited in this study as one of the main barriers to school lunch provision. Important questions that need to be answered include: What are the expected costs of school lunch provision? Will subsidies be available, or will parents be willing to financially contribute to such a program? In this study, parents were open towards the idea of a healthy school lunch and were willing to make a financial contribution; however they deemed the costs should not exceed their current lunch costs, while, simultaneously, demanding that school lunch provision should provide additional options to homemade lunchboxes.

To our knowledge, this is the first study to have explored the perceptions of children, parents and school staff towards the development and implementation of school lunch provision in the Netherlands. Consequently, the results contribute to extant literature on the development of school lunch provision. A further strength of this study pertains to the large number of children that participated, as well as the fact that we consulted schools located in neighborhoods in the Netherlands that comprise people from a wide range of socio-economic backgrounds. One limitation of this study is that there may have been selection bias in our sample. The only schools in Ede that were approached to take part were those that had previously expressed an interest in healthy nutrition. Resultantly, those principals and teachers who were willing to participate may have been inclined to express more positive or elaborate opinions on this topic. Nevertheless, the concerns expressed by our participants are also wholly applicable to schools with less motivated principals and teachers. Moreover, it is important to keep in mind that we asked children, parents and school staff to provide opinions on a concept that they were not previously familiar with.

It is evident that implementing school lunch provision will be a complex undertaking. There are several implementation theories and frameworks that may help in this regard. Although the steps within these different theories differ, there is consensus over the fact that the first step is to establish the needs of the different stakeholders and gain insight into both the barriers and facilitators [33, 34]. It is important to design an implementation plan and align this to the needs of future stakeholders [35, 36]. Our study represents a first step in gaining insight into school lunch provision, and indicates that although it has the potential to be successfully introduced within primary schools, this will only occur if school-based organizational constraints and the needs of all stakeholders are taken into account. Therefore, more research is needed to investigate these aforesaid organizational, financial and logistic issues in greater depth.

### Electronic supplementary material

Below is the link to the electronic supplementary material.

Supplementary Material 1

## Data Availability

The datasets used and/or analysed during the current study are available from the corresponding author on reasonable request.
